# Concurrent assessment of epidemiological and operational uncertainties for optimal outbreak control: Ebola as a case study

**DOI:** 10.1098/rspb.2019.0774

**Published:** 2019-06-19

**Authors:** Shou-Li Li, Matthew J. Ferrari, Ottar N. Bjørnstad, Michael C. Runge, Christopher J. Fonnesbeck, Michael J. Tildesley, David Pannell, Katriona Shea

**Affiliations:** 1 Department of Biology and Center for Infectious Disease Dynamics, The Pennsylvania State University, University Park, PA, USA; 2 State Key Laboratory of Grassland Agro-ecosystems, and College of Pastoral, Agriculture Science and Technology, Lanzhou University, People's Republic of China; 3 US Geological Survey, Patuxent Wildlife Research Center, Laurel, MD, USA; 4 Department of Biostatistics, Vanderbilt University School of Medicine, Nashville, TN, USA; 5 Systems Biology and Infectious Disease Epidemiology Research Centre, School of Life Sciences and Mathematics Institute, University of Warwick, Coventry CV4 7AL, UK; 6 School of Agriculture and Environment, The University of Western Australia (M087), Crawley, WA 6009, Australia

**Keywords:** decision theory, disease management, epidemiological uncertainty, operational uncertainty, optimal control

## Abstract

Determining how best to manage an infectious disease outbreak may be hindered by both epidemiological uncertainty (i.e. about epidemiological processes) and operational uncertainty (i.e. about the effectiveness of candidate interventions). However, these two uncertainties are rarely addressed concurrently in epidemic studies. We present an approach to simultaneously address both sources of uncertainty, to elucidate which source most impedes decision-making. In the case of the 2014 West African Ebola outbreak, epidemiological uncertainty is represented by a large ensemble of published models. Operational uncertainty about three classes of interventions is assessed for a wide range of potential intervention effectiveness. We ranked each intervention by caseload reduction in each model, initially assuming an unlimited budget as a counterfactual. We then assessed the influence of three candidate cost functions relating intervention effectiveness and cost for different budget levels. The improvement in management outcomes to be gained by resolving uncertainty is generally high in this study; appropriate information gain could reduce expected caseload by more than 50%. The ranking of interventions is jointly determined by the underlying epidemiological process, the effectiveness of the interventions and the size of the budget. An epidemiologically effective intervention might not be optimal if its costs outweigh its epidemiological benefit. Under higher-budget conditions, resolution of epidemiological uncertainty is most valuable. When budgets are tight, however, operational and epidemiological uncertainty are equally important. Overall, our study demonstrates that significant reductions in caseload could result from a careful examination of both epidemiological and operational uncertainties within the same modelling structure. This approach can be applied to decision-making for the management of other diseases for which multiple models and multiple interventions are available.

## 1. Introduction

**Figure 1.**
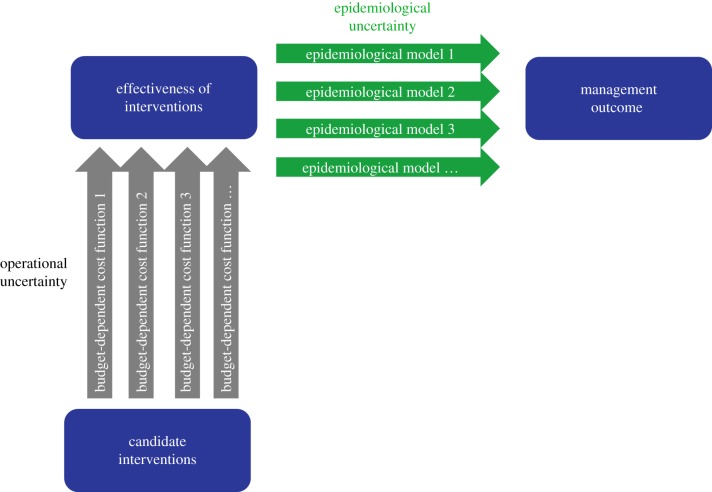
Illustration of the epidemiological and operational uncertainties during the process of assessing the effect of alternative interventions on the final management outcome. Epidemiological uncertainty is represented by a set of alternative models that describe the relationship between biological processes and the outcome of management concern (e.g. reduction of caseload). Operational uncertainty is represented by a set of alternative functions that determine the effectiveness of candidate interventions. (Online version in colour.)

During infectious disease outbreaks, decision-makers seek to identify and implement interventions to most effectively bring the epidemic under control. Decision analysts have identified different sources of uncertainty that can impede decision-making [1–6]. An awareness of such uncertainties, and of how they might affect management outcomes, is essential for planning effective intervention efforts [4,6]. Acknowledging uncertainties is critical to avoid over- or underestimating management effectiveness [7–9]. Studies show that ignoring uncertainties may lead to inefficient or even unsuccessful management [5,10]. During the decision-making process, a critical question that decision-makers face is which candidate intervention, or combination of interventions, is optimal to improve the management outcome. The answers are rarely straightforward; at least two types of uncertainty need to be addressed. The first uncertainty arises from a lack of knowledge about the underlying epidemiological processes, such as the rate of disease transmission and spatial spread [11]. We hereafter refer to this as ‘epidemiological uncertainty', which is also known as model, parametric or structural uncertainty in decision theory (figure 1) [2,5,6]. The second type of uncertainty concerns the magnitude of the effect of any intervention that can be achieved in practice. This type of uncertainty is due to limited information on, for example, logistical constraints, behavioural changes that might arise or compliance with the corresponding intervention during the operational process. We will refer to this type of uncertainty as ‘operational uncertainty', otherwise known as partial control uncertainty or partial controllability in decision theory (figure 1) [2,6].

As a result of limited biological information, especially at the onset of an emerging disease outbreak, epidemiological uncertainties about the structure and parameters of epidemic models may limit reliable prediction of the epidemic trajectory. Quantitative methods provide support for decision-making during epidemics, and mathematical models are increasingly used to understand mechanisms underlying disease transmission and spread, and to evaluate potential strategies for epidemic control [12–14]. As a result of intense scientific interest in the problem, or to account for epidemiological uncertainties, multiple models based on different assumptions and using different modelling approaches are often developed to assist epidemic decision-making; this is particularly the case for severe outbreaks. For example, during the 2001 foot-and-mouth disease outbreak in the UK, multiple models were developed to project the outbreak dynamics and evaluate candidate interventions to assist outbreak management [15–17]. More recently, during the 2014 Ebola outbreak in West Africa, a large set of models was published to address transmission mechanisms and inform decision-making [13,14,18–20]. Using multiple models provides a range of insights and may avoid the bias of a single model. The different predictions and management recommendations resulting from alternative models offer a chance to understand the epidemiological uncertainties and to avoid over- or underestimation of management effectiveness. However, decision-makers need a framework to analyse and integrate multiple models in a way that uses the information they convey about epidemiological uncertainty and supports the decision-making process.

While an increasing number of studies address epidemiological uncertainty in epidemic management [14,21,22], operational uncertainty has received comparatively less attention. Identifying and understanding operational uncertainties, so as to anticipate the logistical and cost constraints affecting candidate interventions, is essential for efficient decision-making. The level of operational uncertainty can vary among individual interventions. For example, while it is relatively straightforward to estimate the extent to which hospitalization can be increased by adding more beds and recruiting more healthcare workers [23], it may be far more difficult to estimate how much community transmission of any disease can be reduced by increasing quarantine or providing household sanitation kits. Moreover, uncertainty is likely to be particularly high when interventions rely on behavioural changes, such as avoiding social contact with infected individuals, through information campaigns [24,25]; behavioural changes in response to an outbreak can alter transmission and potentially reduce epidemic size [25]. Operational constraints on a particular intervention also depend on outbreak settings, including constraints imposed by the prevalent political, cultural and economic conditions [26]. For example, it may be easier to achieve higher hospitalization rates and to reduce hospital transmission in developed rather than in developing countries due to the availability of public health resources.

To identify the optimal intervention for an epidemic management problem, we require an understanding of the inherent uncertainties from both epidemiological and operational perspectives. Ignoring either type of uncertainty may lead to poor recommendations. For example, an epidemiologically effective intervention may not be optimal if it is operationally hard to implement or economically expensive; similarly, a cheap, operationally feasible intervention is not optimal if it is epidemiologically ineffective in controlling the outbreak. Therefore, it is essential to address both types of uncertainties in the same framework. In our previous work [14], we have explicitly addressed how epidemiological uncertainties can affect management recommendations and examined how to use an ensemble of models to identify and resolve the epidemiological uncertainties that most hinder the choice of an optimal intervention. However, further studies to explicitly evaluate operational uncertainties within such epidemic models (i.e. to simultaneously evaluate both epidemiological and operational uncertainties within the same modelling framework) would be highly informative. In the present study, we use Ebola outbreak management to demonstrate the inclusion of both operational and epidemiological uncertainties in an analysis to inform decision-making about management of an epidemic. The analysis is based on a large set of existing models that encapsulate epidemiological uncertainty, in conjunction with an analysis of operational uncertainty. We initially assess their joint importance assuming an unlimited budget. Although we recognize that this is unrealistic, it provides a baseline for comparison with budget-constrained results. In practice, any response to an outbreak always has a budget limit, though the magnitude of this limit and other operational constraints may not be well articulated at the time of response planning [27,28]. We therefore explore the potential cost-effectiveness of interventions at different possible budget levels, to assess the degree to which costs and budget constraints affect intervention rankings. Furthermore, we apply value of information (VoI) analysis [29], which quantifies how much the management outcome could be improved by resolving different sources of uncertainty. VoI can be used to direct new information collection, and to evaluate the potential value of resolving epidemiological and operational uncertainty under different budgetary constraints. Additionally, we discuss the application of the framework developed here across a possible range of public health settings in terms of contingency planning for an outbreak, or as a useful tool during an outbreak.

## 2. Methods

We used the tenets of decision theory to frame our analysis, because we are interested in the applied question of how to inform decision-making regarding management of epidemic outbreaks. The elements of a structured decision-making framework include the objectives, the alternative interventions, and the modelling approach to evaluate the interventions and the sources of uncertainty [11]. We applied these elements in the context of managing a future Ebola outbreak, taking into account both epidemiological and operational uncertainty.

We defined minimizing caseload as the management objective. Other objectives are possible and can have a profound effect on the results [30], but we focused on this one for the purpose of demonstration. We first simulated the caseload under no intervention conditions to provide a baseline. We then identified three classes of alternative sets of management intervention that are widely applied for Ebola control. The first set of interventions involves reducing transmission in the community, which comprises a suite of approaches including rapid contact tracing and case isolation, community awareness campaigns to reduce travel and encourage self-quarantine of sick individuals, provision of household sanitation kits and closing borders. The second set of interventions aims to improve hospitalization, by increasing the proportion of Ebola patients who get hospitalized and reducing transmission within the hospital setting. Improving hospitalization can be achieved in practice by, for example, improving contact tracing and gaining public support to identify and isolate patients, building Ebola treatment centres, and increasing medical supplies and beds. Reducing hospital transmission can be achieved if healthcare personnel use personal protective equipment (PPE) when treating infected cases, and by reducing hospital visits. The third set of interventions involves reducing funeral transmission, which is achieved via safe burial practices.

**Figure 2.**
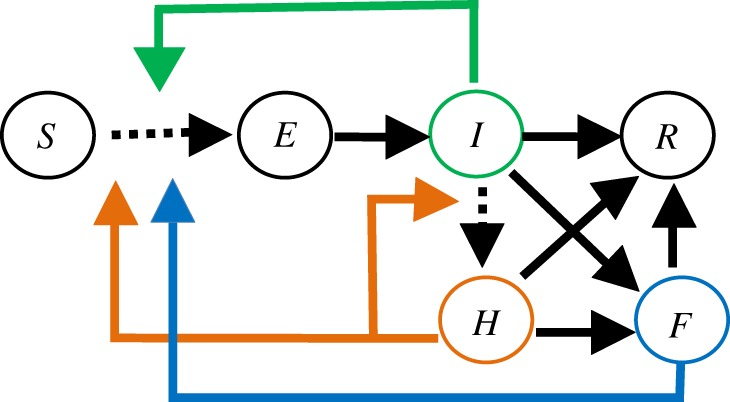
Illustration of an *SEIHFR* compartment model and three widely applied interventions simulated by the model. An *SEIHFR* model includes *S* (susceptible individuals in a population), *E* (exposed individuals), *I* (infectious individuals in the community), *H* (hospitalized individuals), *F* (funerals for infectious individuals who died in the community or hospital) and *R* (individuals removed from the model through either recovery or burial) compartments. The three simulated interventions are reducing transmission in the community (represented by the green arrow), improving hospitalization by increasing the percentage of cases hospitalized and reducing the transmission in hospital (represented by orange arrows) and reducing transmission at funerals (represented by blue arrows). The transitions that are affected by an intervention are shown by arrows with dashed lines.

Our *modelling framework* involved stochastic compartment models, which were used to evaluate the effect of each of the three sets of interventions in achieving the management objective. In the full *SEIHFR* model, individuals in a population progress through susceptible (*S*) to exposed (*E*), infectious (*I*) and hospitalized (*H*) compartments [14]. Infectious individuals (in the community or hospital) finally reach the removed (*R*) compartment either through recovery or death; in the latter case they proceed to the funeral (*F*) compartment before finally being removed from the transmission chain (figure 2). We identified and recoded 37 published compartmental Ebola models. Eight models had all *SEIHFR* compartments represented, seven were *SEIHR* models (excluding an explicit funeral compartment), five were *SEIFR* models (without an explicit compartment for hospitalized individuals) and 17 were *SEIR* models with neither hospital nor funeral compartments [14]. Epidemiological uncertainty is represented by these 37 parameterized Ebola models and the alternative hypotheses implied by each. Detailed information on the variation in key parameters in the 37 models is provided in electronic supplementary material, table S1.

The set of interventions for reducing community transmission was simulated by reducing the transmission coefficient (the rate that disease moves from infected individuals to susceptible individuals in a population; the product of the contact rate and transmission probability given contact) in the community compartments of the model. The set of interventions to improve hospitalization was simulated by simultaneously increasing the proportion of individuals hospitalized and reducing in-hospital transmission. The set of interventions to reduce funeral transmission was simulated by reducing the transmission coefficient in the funeral compartment in the model. The effectiveness of each set of interventions was defined as the percentage change in transmission or hospitalization compared with the baseline. For example, 10% effectiveness in the community transmission intervention was represented by a 10% reduction in the community transmission coefficient in the corresponding simulation. All three sets of interventions can be explicitly simulated in the full *SEIHFR* models, as illustrated in figure 2. For the submodels with simpler structure (for which some compartments were unspecified), the corresponding interventions were implicitly simulated following the approach of Li *et al*. [14], in which the implicit effect of an intervention was calculated via the average proportional contribution of the target transmission to the overall transmission based on the full *SEIHFR* models.

**Figure 3.**
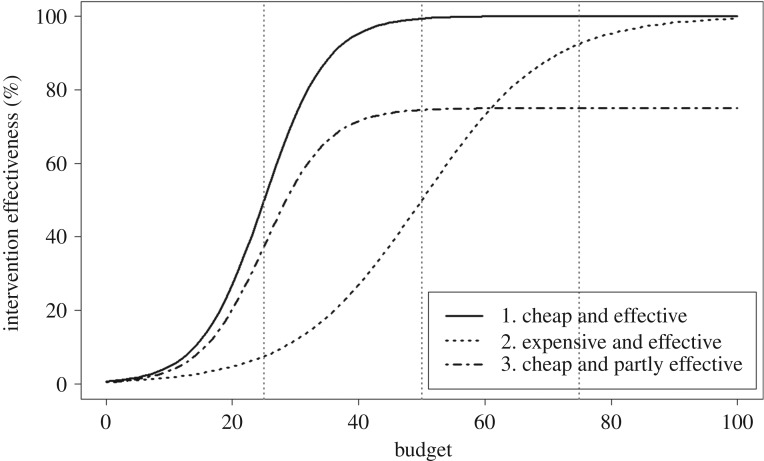
Illustration of three different types of relationship between the expected effect of candidate interventions and the corresponding budget (on a scale of 0–100). The three dotted grey vertical lines at budgets of 25, 50 and 75 represent low, intermediate and high budget levels, respectively.

For each class of intervention, we must assume the effect of the intervention on the corresponding parameters of the dynamic models. Prior to implementation, it may not be possible to know this effect. Hence this uncertainty in intervention effectiveness represents a critical *operational uncertainty*. First, to thoroughly explore operational uncertainties, we simulated each model and intervention combination under the full possible range of intervention effectiveness (from 0 to 100%, with steps of 10%) for each set of interventions without considering the cost. Second, to assess the extent to which costs would affect management recommendations, we explored the effects on caseload projection and intervention rankings of uncertainty in the cost function, combined with three alternative budget levels. We explored three representative cost functions, each of which is plausible for public health management and likely to apply to Ebola interventions (figure 3). The cost functions are assumed to take a logistic form:

2.1 $$f\;(x)=\frac{L}{1+e^{-k(x-x_{0})}},$$

where *x* is the amount invested, in units ranging from 0 to 100, *f*(*x*) is the intervention effectiveness, ranging from 0 to 100%, *L* is the maximum intervention effectiveness, *x*_0_ is the value where *f*(*x*) achieves its midpoint value (i.e. 0.5 × L) and *k* is the steepness of the curve. We adjusted the parameters *L* and *k* for each of the three cost functions to illustrate the differences in their maximum achievable intervention effectiveness (*L* = 75% or 100%) and the increase in intervention effectiveness per unit expenditure (*k* = 0.1 or 0.2). The first cost function, where *L* = 100% and *k* = 0.2, describes interventions that are ‘cheap and effective’. In this scenario, the effectiveness of the intervention increases quickly with increased expenditure, and the effectiveness may approach 100% with further expenditure (figure 3). This type of cost curve might apply to the set of interventions intended to reduce funeral transmission. For example, funeral transmission might be quickly reduced by providing staff and PPE supplies to conduct safe burial, and it may achieve a high effectiveness, approaching 100% reduction in funeral transmission, with completely safe burial practices. The second cost function, where *L* = 100% and *k* = 0.1, represents the interventions that are ‘expensive and effective’; it increases less quickly with increased investment, but the effectiveness may still eventually approach 100%. Such interventions may require a considerable initial investment before generating an obvious effect on outbreak control, but the effectiveness can increase rapidly and achieve a high level once it passes a certain investment threshold (figure 3). This could be the case for the set of interventions for improving hospitalization. For example, the effect of investment in hospital construction cannot be seen until it comes into use; then it increases the proportion of individuals hospitalized. The third cost function, where *L* = 75% and *k* = 0.2, illustrates the interventions that are ‘cheap and partly effective’; the effectiveness increases quickly with initial investment, but saturates at a lower level, i.e. the effectiveness levels off below 100% despite continued investment (figure 3). This might apply to the set of interventions for reducing community transmission. For example, community transmission could be reduced considerably by implementing actions such as educational campaigns or providing household sanitation kits, which are inexpensive, but the impact may stop increasing at some level despite increasing effort. These cost functions represent a range of possible functions; considerable research would be required to estimate the appropriate functional form and parameterization in any real setting. We considered all 27 permutations of the three cost functions across the three sets of interventions (table 1). We did this for each of three budget levels: low (25 out of a total of 100 unit cost), intermediate (50 out of a total of 100 unit cost) and high (75 out of a total of 100 unit cost). We estimated the intervention effectiveness for each of the 27 possibilities (encapsulating our operational uncertainty), projected the caseload under each effectiveness for each of the 37 models (encapsulating our epidemiological uncertainty), and identified the optimal intervention based on the projected caseload for each of the 999 (= 37 × 27) model–cost function combinations under each budget level (table 1; electronic supplementary material, tables S1 and S2).

**Table 1.** Optimal interventions with lowest caseload projection under 27 different cost function combinations for each of 37 Ebola models for a low budget. The three simulated interventions are reducing community transmission (com.), improving hospitalization (hos.) and reducing funeral transmission (fun.). The three simulated cost functions are ‘cheap and effective’ (1), ‘expensive and effective' (2) and ‘cheap and partly effective' (3). The last column and row show the optimal interventions with lowest caseload across models and cost functions, respectively, while the right bottom cell shows the overall optimal intervention across models and cost function combinations. Full information on the caseload and optimal intervention for all 37 models and 27 cost function combinations under low, intermediate and high budget levels is provided in electronic supplementary material, tables S1 and S2.

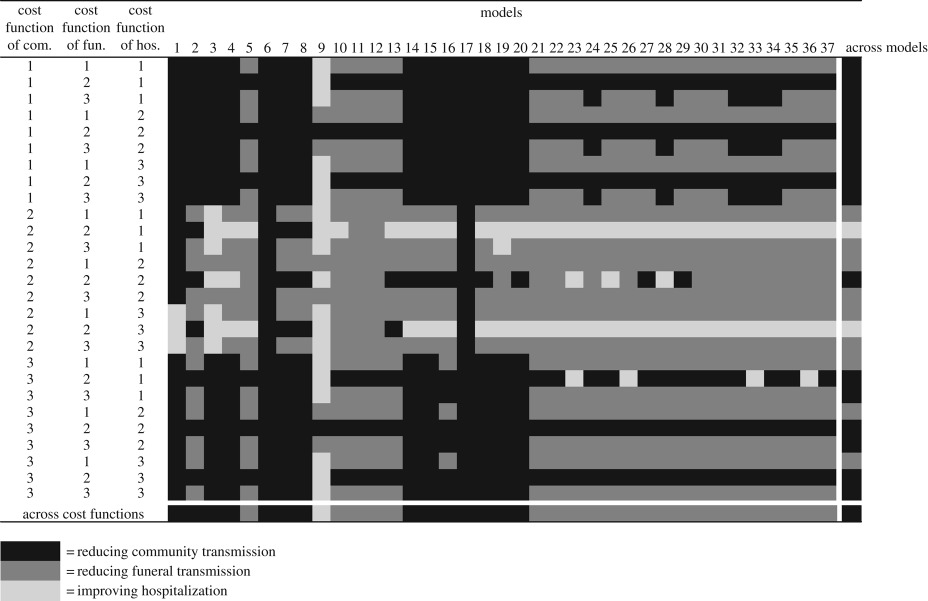

We performed 1000 stochastic simulations for each of the models under all scenarios. Each simulation started with an initial epidemic status of one infectious individual in a population of 10 000 individuals. We assumed a coefficient-of-variation of 0.1 in each transmission coefficient. To ensure equivalent representation, we conducted all simulations in R 3.2.1 [31] using the Gillespie algorithm (with a tau-leap approximation) [32].

VoI analysis quantifies the difference between expected management outcomes achieved via implementing the optimal intervention identified before and after new information is collected, and therefore provides a useful tool to evaluate an information collection strategy [4,11,29]. We conducted VoI analysis to examine how much the management outcome could be improved by new information collection to resolve epidemiological and operational uncertainty [4,7,29]. We first conducted an expected value of perfect information (EVPI) analysis [4,11,29], a common type of VoI analysis, to evaluate the improvement in management outcome from perfect information to resolve all sources of uncertainties. EVPI is calculated as

2.2 $$\mathrm{EVPI}=\;\overset{q}{\underset{i=1}{\sum }}\overset{r}{\underset{\;j=1}{\sum }}p_{i}p_{j}\underset{a}{\min}C_{a,i,j}-\underset{a}{\min}\overset{q}{\underset{i=1}{\sum }}\overset{r}{\underset{\;j=1}{\sum }}p_{i}p_{j}C_{a,i,j},$$

where *C_a,i,j_* represents caseload projected under intervention *a*, model *i* and cost function combination *j*, with *a* = 1, 2, … , *A* (*A* = 3), *i* = 1, 2, … , *q* (*q* = 37) and *j* = 1, 2, … , *r* (*r* = 27). *p_i_* is the weight associated with model *i* (i.e. the prior belief weight that model *i* is the true model; subject to the constraint that the *p_i_* sum to 1), *p_j_* is the weight associated with cost function combination *j* (i.e. the belief weight that cost function combination *j* is the true cost function combination; subject to the constraint that the *p_j_* sum to 1) and min*_a_* indicates the lowest caseload under the optimum intervention. We assigned equal weight to all models and to all cost function combinations; these weights could be updated should evidence (for example, the fit of real-time surveillance data to projections from each model) support a reassessment of model credibility to assign uneven weights [33]. We conducted three separate EVPI analyses assuming low, intermediate and high budget levels. We subsequently conducted expected value of partial information (EVXI) analyses to quantify how much the management outcome could be improved by resolving only epidemiological uncertainty (i.e. identifying the ‘true' model) or only operational uncertainty [4,29]. The EVXI analysis for epidemiological uncertainty as represented by 37 models can be quantified as

2.3 $$\begin{array}{l} \mathrm{EVXI}(\mathrm{epidemiological})\;=\;\sum_{i=1}^{q}p_{i}\underset{a}{\min}\sum_{\;j=1}^{r}p_{j}C_{a,i,j}\\ \;\;-\underset{a}{\min}\sum_{i=1}^{q}\sum_{\;j=1}^{r}p_{i}p_{j}C_{a,i,j}, \end{array}$$

where *n* (*n* = *q* × *r* = 999) model-cost function combinations are grouped into *i* *=* 1 *… q* model sets (*q* = 37). A similar EVXI analysis can be conducted considering the 27 cost function combinations as a representation of operational uncertainty:

2.4 $$\begin{array}{l} \mathrm{EVXI}(\mathrm{operational})\;=\;\sum_{\;j=1}^{r}p_{j}\underset{a}{\min}\sum_{i=1}^{q}p_{i}C_{a,i,j}\\ \;\;-\underset{a}{\min}\sum_{i=1}^{q}\sum_{\;j=1}^{r}p_{i}p_{j}C_{a,i,j}, \end{array}$$

where *n* model–cost function combinations are grouped into *j* *=* 1 *… r* cost function combination sets (*r* = 27). Both EVXI analyses for epidemiological uncertainty and operational uncertainty were also conducted under three budget levels, again assuming that all models were equally weighted, and similarly that each of the three cost-effectiveness curves were equally likely for each intervention.

## 3. Results

**Figure 4.**
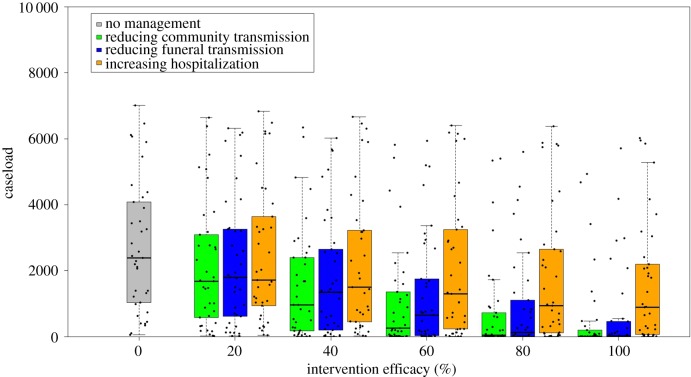
Caseload projected by 37 models under the interventions of reducing community transmission, reducing funeral transmission and improving hospitalization. Black points represent the caseload projections of each model, with 25th, 50th and 75th percentiles marked by the box.

Figure 4 shows the projected caseload of each of the 37 models under each of the three interventions, with effectiveness ranging from 0% to 100% in a simulated population of 10 000 individuals. For a given effectiveness of a particular intervention, caseload varies greatly between models, suggesting a high level of epidemiological uncertainty. Generally, when the intervention effectiveness is the same, caseload is lowest under the intervention of reducing community transmission, intermediate under the intervention of reducing funeral transmission, and highest under the intervention of improving hospitalization. This suggests that reducing community transmission could be epidemiologically most effective in controlling the outbreak. However, the rank of intervention can also be affected by the level of efficacy that is achievable in practice. For example, when each intervention has the same effectiveness level, reducing community transmission is on average more successful than reducing funeral transmission in reducing caseload. However, if reducing community transmission can only achieve a low level of effectiveness, for example 20%, but reducing funeral transmission can achieve 40% or higher, then the intervention of reducing funeral transmission, rather than the intervention of reducing community transmission, ranks as the most successful intervention. Thus, the rank of intervention is not only determined by its epidemiological effect but also the ultimate effectiveness level that can be achieved.

Our results show that the ranking of interventions can also be affected by the relationship between effectiveness and investment (table 1), and by budget constraints (electronic supplementary material, table S2). When the budget is tight, a cheap intervention is most likely to be optimal: reducing community transmission is always optimal provided it is ‘cheap and effective' or ‘cheap and partly effective'; reducing funeral transmission is generally optimal (36 out of 37 models in both cases); and, improving hospitalization is also optimal most of the time (31 out of 37 models in both cases; table 1), assuming both other interventions are expensive. Across models (table 1, last column), reducing community transmission is generally best (in 16/27 cost function combinations). Across cost function combinations (table 1, last row), reducing funeral transmission is generally best (in 22/37 models). Reducing community transmission is optimal across all models and cost function combinations (table 1, last cell). As the budget increases, the ranking of interventions is less affected by the potential cost functions (electronic supplementary material, table S2). For example, with a high budget, the optimal intervention is relatively unchanged across 27 cost function combinations for a given model (electronic supplementary material, table S2—ranking within a column is consistent). Detailed information on caseload and the best-ranked intervention associated with all model, intervention and cost function combinations under each budget level (37 models × 27 cost function combinations × 3 budget levels) is provided in the electronic supplementary material (tables S2 and S3).

To examine how the management outcome can be improved by resolving different sources of uncertainty, we conducted EVPI and EVXI analyses based on electronic supplementary material, table S3. Overall, our EVPI results showed that, by resolving all uncertainties in both epidemiological and operational settings, caseload could be reduced by 18.7%, 41.5% or 57.3% under low, intermediate and high budgets, respectively (table 2). Such high EVPI values suggest that reducing uncertainty surrounding the decision-making process is highly worthwhile, and is especially beneficial when budgets are large. The EVXI analysis shows that reducing epidemiological uncertainty (as represented by the 37 models) is more beneficial under moderate and high budget levels than under low budget level (33.6% and 52.6% versus 10.1% reduction in caseload; table 2). On the other hand, the EVXI analysis of operational uncertainty shows that while resolving uncertainty about the cost function only improves the management outcome by 4.8% under high budget conditions, the effect is higher when the budget is tight, with the management outcome improved by 11.3% and 13.1% under intermediate and low budget levels, respectively (table 2). Comparison of the EVXI analyses of epidemiological uncertainty versus operational uncertainty showed that, when the budget level is low, it is almost equally important to resolve epidemiological and operational uncertainties (10.1% versus 11.3% reduction in caseload; table 2), while resolution of epidemiological uncertainties becomes relatively more beneficial under high budget level conditions (52.6% versus 4.8%; table 2).

**Table 2.** The value of resolving epidemiological and operational uncertainty in an epidemic Ebola setting. Expected value of perfect information (EVPI) represents the improvement in management outcome in terms of reduction in caseload by resolving all sources of uncertainty perfectly (see electronic supplementary material, table S3 for calculations). Expected value of partial information (EVXI) represents the improvement in management outcome by resolving a particular source of uncertainty, specifically epidemiological or operational uncertainty.

	budget		
	low	medium	high
minimum of the average caseload across models and cost functions	1829	881	621
average of the lowest caseload across models and cost functions	1486	515	265
EVPI	343	366	356
improvement in management %	18.7%	41.5%	57.3%

average of the lowest caseload across models	1645	585	294
EVXI	185	296	326
improvement in management %	10.1%	33.6%	52.6%

average of the lowest caseload across cost functions	1623	765	590
EVXI	206	115	30
improvement in management %	11.3%	13.1%	4.8%

## 4. Discussion

During the epidemic decision-making process, a key issue is the identification of interventions that will most effectively bring an outbreak under control. As disease outbreaks are often unexpected, they are also usually associated with significant uncertainty about epidemiological processes, and about how successful interventions will be. At one extreme, for an entirely novel pathogen, uncertainty about the dynamics of the disease (i.e. epidemiological uncertainty) needs to be addressed first, because potentially useful interventions cannot even be identified without at least some knowledge of the disease. At the other extreme, a common disease re-emerging in a new context might mean that the epidemiology is fairly well known, and the information most lacking may be on intervention effectiveness under the new conditions (i.e. the main uncertainties are operational). For a re-emerging disease, however, epidemiological knowledge of transmission dynamics may exist, but outbreak characteristics (such as the basic reproduction number (*R*_0_) and clinical severity) may differ in new settings. In general, in most outbreak settings, such as the 2014 West African Ebola outbreak examined here, there are both epidemiological and operational uncertainties that impede decision-making. In such situations, it will be important to assess these two forms of uncertainty concurrently within a common framework.

Classic analyses of management examine alternative interventions in a single model and rank them by their effect on the outcomes of interest. With the rapid development of quantitative forecasting tools for epidemics, coupled with increased data availability, multiple models are commonly built to project disease trajectories and inform public health policy [14]. Multiple alternative models provide an opportunity to improve the representation of uncertainty underlying the epidemic process, and avoid the bias that may arise from using a single model [34,35]. Understanding and quantifying the epidemiological uncertainties represented by multiple models is thus critical to identify the optimal interventions in epidemic management. In the current study, we explored an ensemble of models to assess the effects of epidemiological and operational uncertainties on management recommendations. Rather than trying to select a single ‘best' model and use it to evaluate candidate interventions, this study addresses how the uncertainty, as expressed in the multiple existing published models, affects the choice of action. As illustrated in figure 2, each of the 37 models can be envisioned as a subset of a synthetic model, reflecting expert understanding of the model structure and parameters. Therefore, by evaluating each candidate intervention over the whole set of models, our results can be used to fully evaluate the effect of this epidemiological uncertainty. Our study shows that the magnitude of caseload projections varies substantially across models (figure 4), suggesting a high level of epidemic uncertainty and that management outcomes based on interventions assessed in any single model could be overly optimistic or pessimistic. Our EVXI analysis further showed that—in the context of the 37 Ebola models—resolving epidemiological uncertainty could improve the management outcome by 52.6% on average, suggesting that new information to improve the understanding of the epidemic process (that is, being able to identify the best model to project the epidemic dynamics) could potentially reduce disease burden by half.

We further find that ignoring operational uncertainty may affect the ranking of alternative interventions and lead to suboptimal management decisions. Our EVXI analysis shows that new information to resolve the operational uncertainty in intervention effectiveness could improve the management outcome by up to 13.1%, meaning 3753 fewer cases for the 2014 Ebola outbreak. This suggests that previous studies failing to include operational uncertainty [11,14] underestimate the value of operational research to support decision-making. One of the reasons for failing to include operation uncertainty in previous studies is that evaluating the intervention effectiveness that can be achieved under logistical constraints is challenging, because the levels of uncertainty about logistical constraints may differ from intervention to intervention, and the operational uncertainty may be more difficult to ascertain for one intervention than for another. Quantifying the caseload reduction that is affordable given the intervention costs is another challenge to the resolution of operational uncertainty, and it requires including the cost functions themselves, and uncertainty about the cost functions. While a failure to consider cost functions means that one might choose effective but expensive interventions, ignoring uncertainty in the cost function may lead to suboptimal decision-making by over- or under-estimating the management outcome. As demonstrated in our study, the optimal intervention varies under different intervention-cost function scenarios (table 1; electronic supplementary material, table S2). Estimating the cost-effectiveness function for a particular intervention in practical management can be challenging, because resources are often directly or indirectly shared by different interventions, and the effects of different interventions commonly interact.

Information about the effectiveness that a particular intervention can achieve is often limited in epidemiological studies (but see [36] on vaccine effectiveness), due to challenges in untangling the joint effects from different interventions, for which high-resolution data are required (e.g. [37]). Studies to provide deeper insights into the logistical constraints, and cost-effectiveness information for individual interventions would facilitate epidemic decision-making. From the standpoint of policy-makers, information that anticipates how much the management outcome could be improved by per unit investment would help managers to determine the optimal intervention and plan management efforts. Input from economists and operations researchers in terms of economic data collection and modelling would also allow a better description of the relationship between the cost and the effectiveness of particular interventions. We argue that the best outcomes will be achieved by integrating the cost-effectiveness functions and the simulations of effectiveness-management outcomes via epidemic models into an overall cost-management outcome framework as illustrated by this Ebola case study. Such a framework requires the joint efforts of epidemiologists and economists.

Our results show that both epidemiological and operational uncertainties would have affected the choice of intervention in the context of the 2014 Ebola outbreak, and that the value of new information collection, which could allow an improvement in management outcome of up to 57.3%, is generally high. Additional information to resolve epidemiological uncertainty could be obtained through real-time surveillance of epidemiological processes (e.g. mortality rates, transmission), while monitoring of intervention activities and their effectiveness would help to reduce operational uncertainty (e.g. [38]). Our EVPI analysis also shows that the overall value of information increases with increasing budget availability, suggesting that such new information collection could be especially beneficial under high or unlimited budget conditions. Furthermore, our EVXI results show that the exact benefit of resolving a particular source of uncertainty could be affected by budget conditions. For example, resolving epidemiological uncertainty yields the most improvement in management outcome when the budget level is high, while resolving operational uncertainty yields is relatively more important to management outcome under low to moderate budget levels. The low, intermediate and high budget level values in the current study were used to illustrate the dependence of management recommendations on budget circumstances. In practice, the budget for Ebola control during the 2014–2016 outbreak was itself highly uncertain, and changed over time. The WHO asked the international community to fill a $71 million gap when issuing the initial regional plan on 31 July 2014. The cost estimates then rose to $490 million on 28 August and $988 million in a UN appeal in mid-September [27,28], as the outbreak worsened and it became apparent that a larger response was necessary to both control further spread and recover from its impacts. In November 2014, the Obama administration asked US Congress to approve $6.18 billion in funding to fight Ebola [39], and the US Congress passed the president's emergency appropriation of $5.4 billion.

We have not investigated whether our results extend broadly to other disease outbreak settings, but we conjecture that they may. For example, for severe disease outbreaks of significant public health concern, like Ebola or Zika, spending may exceed initial budgets in the pursuit of a critical management objective (as seen in the case of polio eradication); therefore research could focus on the resolution of uncertainties surrounding the epidemiological processes. For other types of outbreaks, such as some agricultural diseases, budget constraints may play a more important role in decision-making. We offer our conjecture as a hypothesis for future study. Although we cannot make remarks regarding our conclusions' generality, the approach developed and applied to identify the uncertainties that most impede decision-making in the context of disease outbreaks is general.

We have illustrated the joint consideration of operational and epidemiological uncertainty as demonstrated in our retrospective study. This approach can also be used prospectively to guide information collection, preparedness planning and inform decision-making for future outbreaks. By analysing the potential uncertainties *before* an outbreak occurs, we may be able to pinpoint key epidemic processes and parameters, important constraints on interventions, or operational limitations which might alter the ranking of interventions, and therefore management decisions [4,11]. For example, estimating the potential range of the transmission coefficients of different transmission sources, plus a corresponding parameter sensitivity analysis, would help to rank the interventions targeting different sources of transmission. Similarly, identification of approximate functional forms for intervention costs, and of key fixed costs, both of which clearly could change outcomes for our Ebola decisions, might be possible before (for better-known diseases) or in the early stages (for novel diseases) of an outbreak. Similar insights were found in a VoI analysis of vaccination for foot-and-mouth disease in the UK; vaccination capacity was a key uncertainty in this situation and could be addressed via pre-outbreak contingency planning [40]. An enhanced prospective approach is also possible. Because not all published models were designed to be comprehensive (i.e. to evaluate all intervention options), the models might not represent the full range of epidemiological uncertainty that does, in fact, exist. An exciting future extension would be to engage multiple modelling groups to develop their models with the same set of outputs and the same set of alternatives in mind (i.e. by careful *a priori* framing of the decision problem). This would do a better job of representing the full range of epidemiological uncertainty that exists.

Valuable insights may also be obtained *during* an outbreak, both in terms of identifying unknown information which is nevertheless safe to ignore versus information that is most important to gather as the outbreak proceeds. As time during an outbreak is limited and valuable, and there is an opportunity cost associated with learning, it is important to prioritize the collection of information related to the most important uncertainties; information is more valuable if it might change the management decision [29]. Epidemic and operational uncertainties can be reduced in real time during an outbreak as new models to understand outbreak dynamics are developed, as the understanding of the outbreak itself evolves [33], and as information on intervention effectiveness becomes available. For a relatively long outbreak, such as the 2014 Ebola outbreak in West Africa, there may be opportunities for real-time learning and adaptation of the management strategies as new information on epidemiological and operational uncertainties are collected [11,20].

Overall, efficient epidemic decision-making necessitates accounting for the uncertainty underlying both disease dynamics and intervention efficacy. Our work illustrates a framework to explore the epidemiological and operational uncertainties on the same platform to facilitate decision-making. There is a need for increased interaction between the communities that study the epidemiological consequences of interventions (often housed in public health or dynamical systems fields) and the communities that study the operational and logistical dynamics of the implementation of interventions (often housed in operations research, sociology, health systems administration and communications). These two disciplines have largely been developing independently; bringing them together will translate into improved management outcomes. This approach should be applicable to other epidemic management scenarios where multiple models or multiple alternative representations of uncertainty are available.

## Supplementary Material

Table S1

## Supplementary Material

Table S2

## Supplementary Material

Table S3

## Supplementary Material

Ebola_Uncertainty.r

## Data accessibility

Data and code supporting the present paper are provided as electronic supplementary material.
