# Supplementary material for: Concurrent assessment of epidemiological and operational uncertainties for optimal outbreak control: Ebola as a case study
Source: Proc Biol Sci. 2019 Jun 19;286(1905):20190774. doi: 10.1098/rspb.2019.0774 (PMC6599986; doi:10.1098/rspb.2019.0774)
Supplement: Table S2 [file rspb20190774supp2.docx]

Table S2 Optimal interventions with lowest caseload projection under 27 different cost function combinations for each of 37 Ebola models for low, intermediate and high budget levels. The three simulated interventions are reducing community transmission (Com.), improving hospitalization (Hos.) and reducing funeral transmission (Fun.). The three simulated cost functions are "cheap and effective" (1), "expensive and effective" (2), and "cheap and partly effective" (3). Cells filled with dark gray, intermediate gray and light gray color indicate the lowest caseload under the optimal intervention of reducing community transmission, reducing funeral transmission, and improve hospitalization, respectively. The last column and row in each budget panel show the optimal interventions with lowest caseload across cost function models and combinations, respectively, while the right bottom cell in each budget panel shows the overall optimal intervention across models and cost function combinations.
